# Clinical Characteristics and Healthcare Resource Utilization among Patients with Obstructive Hypertrophic Cardiomyopathy Treated in a Range of Settings in the United States

**DOI:** 10.3390/jcm11133898

**Published:** 2022-07-04

**Authors:** Michael Butzner, Ethan Rowin, Amin Yakubu, Josiah Seale, Laura A. Robertson, Phil Sarocco, Martin S. Maron

**Affiliations:** 1 Cytokinetics, Incorporated, Health Economics and Outcomes Research, 350 Oyster Point Blvd, South San Francisco, CA 94080, USA; pwsarocco@gmail.com; 2 Hypertrophic Cardiomyopathy Center and Research Institute, Division of Cardiology, Tufts Medical Center, 860 Washington St Building, Boston, MA 02111, USA; ethan.rowin@lahey.org (E.R.); martin.maron@lahey.org (M.S.M.); 3 Genesis Research, 5 Marine View Plaza, Hoboken, NJ 07030, USA; amin@genesisrg.com (A.Y.); josiah@genesisrg.com (J.S.); 4 Cytokinetics, Incorporated, Clinical Research, 350 Oyster Point Blvd, South San Francisco, CA 94080, USA; laura.robertson.md@gmail.com

**Keywords:** obstructive hypertrophic cardiomyopathy, healthcare resource utilization, electronic medical records, real-world

## Abstract

Obstructive hypertrophic cardiomyopathy (oHCM) has been studied primarily in comprehensive centers of excellence. Broadening the understanding of patients with oHCM in the general population may improve identification and treatment in other settings. This retrospective cohort study identified adults with oHCM from a large electronic medical record database comprising data from 39 integrated delivery networks (IBM Explorys; observational period: January 2009–July 2019). Clinical characteristics, healthcare resource utilization (HCRU), and outcomes were reported. Of 8791 patients, 53.0% were female and the mean index age was 61.8 years. Cardiovascular drugs prescribed included beta-blockers (80.5%), calcium channel blockers (46.0%), and disopyramide (2.4%). Over time, heart failure, atrial fibrillation, and ventricular arrhythmias increased. Surgical procedures included septal myectomy (22.0%), alcohol septal ablation (0.6%), and heart transplantation (0.3%). Implantable cardioverter defibrillators were present in 11.2% of patients. After initial septal reduction therapy (SRT), HCRU increased and 550 patients (27.7%) required a reintervention. Of the overall group, 2.7% experienced sudden cardiac arrest by end of study. In conclusion, this cohort of patients with oHCM had guideline-recommended drug therapy and procedures. Despite this, heart failure, atrial fibrillation, and ventricular arrhythmias increased, and more than a quarter of patients undergoing SRT required reintervention. These unresolved issues emphasize the unmet need for new, effective therapies for patients with oHCM.

## 1. Introduction

Hypertrophic cardiomyopathy (HCM) is a complex cardiac disease with a highly variable clinical profile [1]. Patients with left ventricular outflow tract obstruction, also known as obstructive HCM (oHCM), account for nearly two-thirds of patients with HCM and have a significant burden of comorbidities, including hypertension, heart failure, and atrial fibrillation [2]. The primary treatment for oHCM is pharmacotherapy, including beta-blockers, calcium channel blockers (CCBs), or disopyramide. If symptoms persist or the obstruction worsens on drug therapy, invasive septal reduction therapies (septal myectomy or transcatheter alcohol septal ablation) may be indicated. Implantable cardioverter defibrillator placement may be recommended based on sudden cardiac death risk. In rare cases of end-stage heart failure, heart transplantation may be required [3].

Previous investigations of patients with HCM from single HCM centers of excellence, representing selected referral populations, have shown that patients with HCM benefit from contemporary treatments [3,4,5,6,7,8,9]. The clinical profile and management of patients with oHCM have been well characterized within established HCM cohorts; however, there is limited real-world evidence on the clinical profile of patients with oHCM in the general community practice across the United States, and the management of patients in the community remains unresolved [10,11,12,13,14,15,16,17,18,19,20]. Broadening the understanding of the profile of patients with oHCM in the general US population may improve screening, identification, and treatment. Therefore, we sought to characterize the clinical characteristics, healthcare resource utilization (HCRU), treatment, and management patterns of patients with oHCM in the United States by utilizing a large, real-world electronic medical records (EMR) database.

## 2. Materials and Methods

### 2.1. Study Design and Population

**Figure 1:**
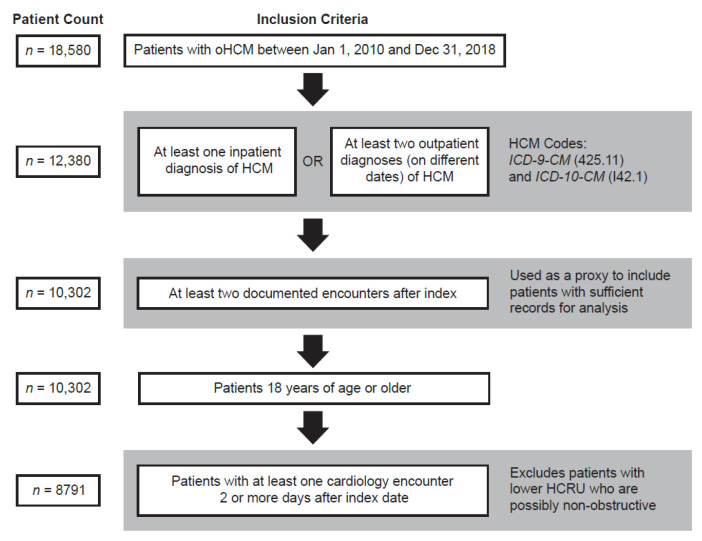
Flow chart of oHCM cohort attrition. HCM: hypertrophic cardiomyopathy; HCRU: healthcare resource utilization; ICD-9-CM: International Classification of Disease, Ninth Revision, Clinical Modification; ICD-10-CM: International Classification of Disease, Tenth Revision, Clinical Modification; and oHCM: obstructive hypertrophic cardiomyopathy.

This retrospective observational study included data from the 10-year period between 2 July 2009 and 2 July 2019. We identified patients who had received a diagnosis of oHCM between 1 January 2010 and 31 December 2018 (Appendix A). The first diagnosis date served as the index date for the analysis, and at least two encounters were required within 6 months post-index date as a proxy for patient engagement (Figure 1). After this time, a minimum period of enrollment was not required. Patient health outcomes were assessed at three follow-up dates: 12 months after the index date, 24 months after the index date, and at the end of the study period. Patients meeting criteria for inclusion were included in all follow-up assessments. For patients with an index diagnosis in 2018, we assessed their follow-up until 2 July 2019 and recorded that as the end of the study period. Adult (≥18 years of age) patients were required to have either one inpatient or two outpatient diagnoses for oHCM (*International Classification of Disease, Ninth Revision, Clinical Modification* [ICD-9-CM] diagnosis code 425.11 or *International Classification of Disease, Tenth Revision, Clinical Modification* [ICD-10-CM] diagnosis code I42.1) within the study period. To ensure the study included only patients with a full patient journey, we required patients to have ≥1 cardiology-related physician specialty encounter ≥2 days after index (Figure 1). Records from the 6 months before the index date were used to confirm the patient’s age, body mass index (BMI), and other demographics.

### 2.2. Data Source

IBM Explorys (IBM, Armonk, NY, USA) is a commercially available database containing longitudinal EMR data of approximately 63 million patients from 39 integrated delivery networks composed of nearly 360 hospitals and 920,000 providers. The database contains information regarding health care provided in the inpatient, ambulatory, emergency, and post-acute settings and includes diagnoses, procedures, medications, laboratory test results, patient-reported outcomes, vital signs, BMI, encounter-level information, providers, and other clinical and operational data. Diagnosis data are coded using ICD-9-CM, ICD-10-CM, and Systematized Nomenclature of Medicine (SNOMED), and procedures are coded using the current procedural terminology, the healthcare common procedure system, SNOMED, or the ICD-9/ICD-10 procedure coding system. All database records are anonymized and fully deidentified, and thus this study did not require approval from an institutional review board.

### 2.3. Study Measures and Data Analysis

Appendix B includes variables that were captured in our data analysis for patients with oHCM. Demographic characteristics included age, sex, ethnicity, race, insurance type, and geographic region. We captured the usage of drug treatments using a string search of the generic and brand names. Comorbidities and HCM-related outcomes were captured using ICD-9-CM/ICD-10-CM codes. Sudden cardiac arrest was captured using the codes ICD-9: 427.5 and ICD-10: I46.x, which encompass all settings of care. We used a combination of Current Procedure Terminology, ICD-9/ICD-10 Procedure Coding System, and SNOMED codes to ensure that all procedures were captured. Procedures were divided into two categories: diagnostic and surgical procedures for oHCM treatment. For patients who underwent septal reduction therapy, radiofrequency ablation, pulmonary vein ablation, and other ablation, we also report new-onset atrial fibrillation, defined as the first recorded >3 months after the procedure. Descriptive analyses for study measures were performed to obtain the means, medians, standard deviations, and interquartile ranges. Categorical variables were summarized using frequency and percentage of patients. All analyses were performed using R 3.5 (The R Foundation, Vienna, Austria) or Microsoft Excel^®^ 2013 (Microsoft Corporation, Redmond, CA, USA) [21,22].

## 3. Results

### 3.1. Baseline Characteristics

**Figure 2:**
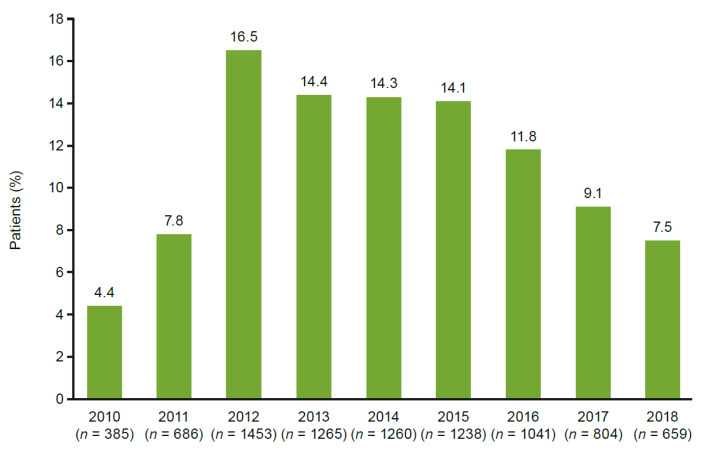
Patients with index diagnosis during each study year.

The final oHCM cohort meeting study criteria included 8791 patients (Figure 1). Demographics are shown in Table 1. About half the group (53%) were female, and the mean age was 61.8 years. The majority of patients were White (81.2%), followed by African American (13.2%). Mean BMI at baseline was 30.4 kg/m^2^. The majority of patients resided in the Midwest (54.9%), followed by the South (27.4%), the Northeast (8.9%), and the West (8.0%). Private insurance holders comprised 59.0% of the cohort, with 46.3% covered by Medicare and 8.1% by Medicaid. The index date for oHCM appeared fairly evenly distributed, albeit with a slightly lower proportion of patients identified during 2010 (Figure 2).

### 3.2. Outcomes and Healthcare Resource Utilization

**Figure 3:**
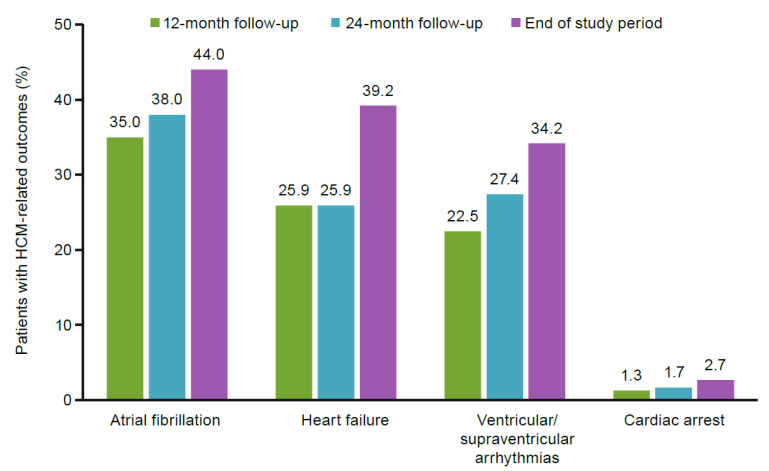
Patients with HCM-related outcomes. HCM: hypertrophic cardiomyopathy.

Table 2 shows diagnosis, hospitalization, and comorbidities. Diagnostic procedure rates had increased by the end of the study period: cardiac imaging was recorded for 87.4% of the cohort, followed by electrocardiography (73.4%), exercise stress testing (32.0%), and coronary angiography (14.1%). Approximately one-third of patients (33.1%) had a record of hospitalization over the study period. Comorbidities increased over the study period, including hypertension (79.8%) and conduction disorders (30.3%). All drug therapy prescription rates increased from the 12-month follow-up to the end of the study period (Table 2). Beta-blockers were prescribed the most (80.5%), and 46.1% of patients were on CCBs. Disopyramide was prescribed to 2.4% of patients by the end of the study period. Of the surgical procedures, more patients underwent a septal myectomy (22.0%) than any other procedure. Implantable cardioverter defibrillators were present in 11.2% of patients, and only 27 patients (0.3%) had a heart transplantation by the end of the study period. HCM-related outcomes also increased, with 44.0% of patients having atrial fibrillation, 39.2% heart failure, and 34.2% ventricular or supraventricular arrhythmias, and 2.7% of patients experienced sudden cardiac arrest at the end of the study period (Figure 3).

### 3.3. Septal Reduction Therapy

**Figure 4:**
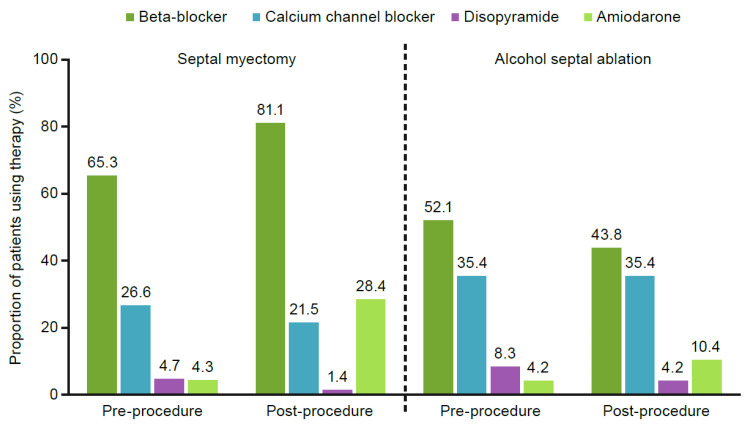
Drug therapy usage before and after septal myectomy or alcohol septal ablation.

**Figure 5:**
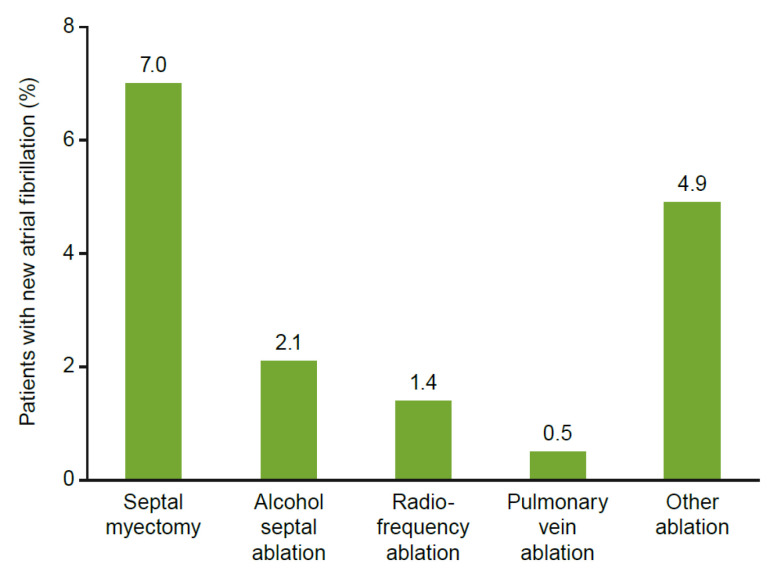
New-onset atrial fibrillation after procedures. New-onset atrial fibrillation is defined as >3 months after procedure. Percentages indicate the proportion of all patients who received a procedure.

**Figure 6:**
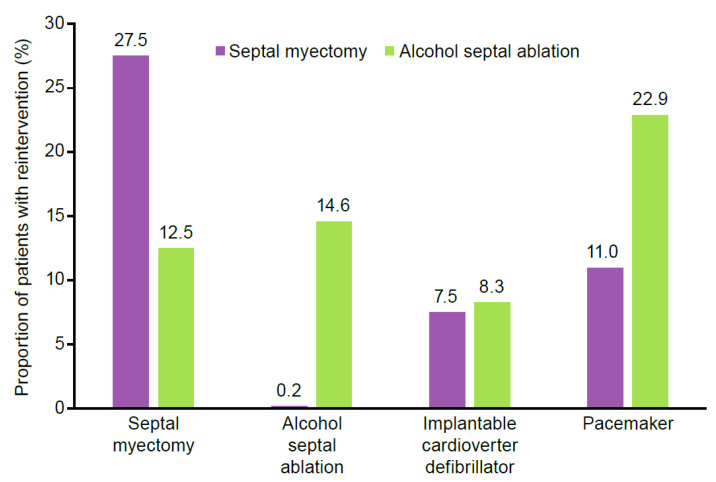
Reintervention rates following initial septal reduction therapy.

Patients who underwent a septal myectomy or alcohol septal ablation (*n* = 1985, 22.6%) had increased HCRU after the procedure. Before the procedure, the mean (±standard deviation) number of HCM-related visits was 5.0 ± 5.2, compared with 6.6 ± 8.6 for post-procedure visits. Figure 4 reports the rates of drug therapy use before and after a myectomy or alcohol septal ablation. After undergoing a septal myectomy, the percentage of patients utilizing drug therapies increased: beta-blockers from 65.3% to 81.1%, and amiodarone from 4.3% to 28.4%. Disopyramide and CCBs reduced in usage after septal myectomy, and 226 (15.9%) patients developed new-onset atrial fibrillation after myectomy or any type of ablation, primarily septal myectomy (*n* = 135, 7%) (Figure 5). After initial septal reduction therapy, 550 patients (27.7%) had a reintervention, with the highest proportion requiring a second septal myectomy (27.5%) (Figure 6).

## 4. Discussion

The purpose of this study was to characterize the clinical characteristics, HCRU, and outcomes of patients with oHCM in the United States by utilizing a large, real-world EMR database. These data showed that patients with oHCM were generally over the age of 60 years and were predominantly White and non-Hispanic. Patients experienced guideline-recommended use of drug therapy, and septal myectomy was the most performed procedure, whereas alcohol septal ablation was infrequent in this cohort. After initial septal reduction therapy, 27.7% had a reintervention. Approximately one-third of patients had a record of hospitalization at least once during the study follow-up, and the percentage experiencing sudden cardiac arrest was low.

The Explorys database contains a vast amount of data on approximately 63 million patients from 39 integrated delivery networks composed of nearly 360 hospitals and 920,000 providers. A major advantage of this real-world EMR database is the inclusion of comprehensive, longitudinal clinical variables that allow for the analysis of health conditions over an extensive observational period. These data cover all regions in the US across multiple patient care settings, including community-based clinics and HCM centers of excellence. It also includes detailed clinical data, including inpatient, ambulatory, emergency, and post-acute settings, and includes diagnoses, procedures, medications, laboratory test results, patient-reported outcomes, vital signs, encounter-level information, providers, and other clinical and operational data. The results of this exploratory analysis set the stage for future analysis to build upon this study by exploring these fields using large EMR data.

The mean age of the cohort was 61.8 years, which is higher than recent studies from HCM centers [2,4,6,7,9] and one study analyzing a large proprietary claims database [23]. Older age in our cohort of real-world patients could be a result of delayed diagnosis due to the underrecognized nature of HCM in clinical practice. The use of recommended first-line drug therapies, including beta-blockers and CCBs, was corroborated in this analysis but at greater rates compared with established HCM cohorts [2,4,6,7,9]. Only 25.4% of patients with oHCM remained off beta-blockers, CCBs, or disopyramide during the study period. These data also present new insights into the use of surgical treatments for oHCM, including septal reduction therapy. Septal myectomy was the most commonly performed surgical procedure. After myectomy or alcohol septal ablation, patients had similar or increased use of medication, and some patients had reintervention, possibly indicating unresolved issues not seen to this extent in previous studies. We speculate that the reason for increased reintervention, along with increased drug therapy and atrial fibrillation following septal reduction therapy, is due to the inherent complexity of the surgical procedure in a variety of care settings that extends beyond HCM specialty centers.

Contrary to previous investigations, comorbidities in this cohort were remarkably higher than previously reported at HCM centers [2,4,6,7,9]. However, rates of comorbidities, drug therapy, and HCM-related outcomes were similar in comparison to recent investigations using IBM MarketScan Commercial and Medicare claims data [24,25]; however, our study utilized IBM EMR data. Regardless, these studies [24,25], like our results, highlight the differences in real-world HCRU and outcomes among patients with oHCM compared with HCM centers. This may reflect differences in disease management between community-based practice and specialized HCM centers of excellence; however, the high rates of comorbidities may also be partly due to a lack of specific and appropriate use of ICD codes. For example, it is unlikely that the high rates of hypertension reflect the true rates found in an oHCM cohort and may be due to the misclassification of oHCM as hypertension, or a need to require multiple diagnosis codes to identify true cases of hypertension, which would exclude misclassifications of comorbidities over a patient’s full clinical journey. Lastly, the rate of sudden cardiac arrest in our analysis was low, at 2.7%, yet is likely to reflect the true prevalence as this outcome was captured across all settings with specific ICD codes. Similar rates of sudden cardiac death have been reported in previous real-world studies using large healthcare claims data, [26,27], and from studies of patients in specialty HCM centers and the general community practice [4,10,11,28,29]. Furthermore, low rates of sudden cardiac arrest in this real-world population concur with these previous studies that HCM is a disease characterized by low mortality.

This is the first study to examine a national sample of patients with oHCM in a large EMR clinical dataset across the United States. Patients with oHCM experienced guideline-recommended use of drug therapy, and surgical treatments appeared to appropriately reflect the treatment of patients with oHCM who have disease progression resistant to drug therapies. Despite appropriate treatment, there was an increase in heart failure, atrial fibrillation, and ventricular arrhythmias over the study period. Over a quarter of patients had a reintervention after initial septal reduction therapy. Taken together, our results provide new insights for clinicians and decision makers, suggesting that there is an unmet need for more effective therapies for oHCM. This hypothesis needs further evaluation, and future research utilizing real-world data to evaluate outcomes in patients with oHCM from multiple care settings that are generalizable to the US population could help to provide this information. In that regard, the current study provides a framework for future analyses utilizing large EMR databases to evaluate oHCM.

### Limitations

There are several limitations to our study, most pertaining to the use of large EMR data. First, oHCM diagnosis was based on ICD-9 and ICD-10 diagnosis codes, which may not provide exact confirmation without patient anatomic or genetic data. This limitation was mitigated by requiring eligible patients to have at least one inpatient diagnosis or two outpatient diagnoses for HCM on separate dates. There are no established methods for identifying patients with oHCM in large clinical EMR databases. Therefore, our study relied on an algorithm that focused on excluding false positives (patients with non-oHCM) at the expense of possibly excluding false negatives. This conservative approach was chosen to ensure that all patients with non-oHCM were definitively excluded, but this method could have potentially excluded certain patients with oHCM. Second, the geographic skew of the cohort to the Midwest may be due to the underlying database structure as HCM is not known to be more common in one region. Third, providers in this cohort primarily used the general ablation code (other unspecified ablation category), and this may have contributed to a disproportionately lower rate of HCM-related alcohol septal ablation in this cohort versus much higher rates seen in HCM centers of excellence [5,6,9,30,31,32,33]. Fourth, due to the inherent nature of large EMR data, we were unable to collect mortality and deep-level clinical data that are common at site level (left ventricular outflow tract gradient, maximal wall thickness, left ventricular ejection fraction, etc.) or information on healthcare costs.

## 5. Conclusions

In summary, patients with oHCM had guideline-recommended use of drug therapy and procedures, and a low percentage of patients experienced sudden cardiac arrest. However, despite the use of contemporary treatment strategies for oHCM, there was an increase in heart failure, atrial fibrillation, and ventricular arrhythmias, and after SRT, over a quarter of patients required reintervention. The identification of these unresolved issues provides new information for clinicians and decisions-makers and emphasizes the importance of new, effective therapies to address this unmet clinical need in patients with oHCM.

## Figures and Tables

**Table 1:** Baseline demographic information.

Demographic Category	Patients with oHCM (*n* = 8791)
Sex, *n* (%)	
Male	4134 (47.0)
Female	4657 (53.0)
Age at index, years	
Mean ± SD	61.8 ± 15.0
Median (IQR)	63.0 (21.0)
Age categories, years, *n* (%)	
18–39	773 (8.8)
40–54	1774 (20.2)
55–64	2101 (23.9)
65–74	2222 (25.3)
75+	1921 (21.9)
Ethnicity, *n* (%)	
Hispanic	300 (3.4)
Non-Hispanic	7769 (88.4)
Other	722 (8.2)
Race, * *n* (%)	
African American	1164 (13.2)
Asian	111 (1.3)
White	7138 (81.2)
Hispanic Latino	33 (0.4)
Other	734 (8.3)
Insurance type, * *n* (%)	
Medicare	4070 (46.3)
Medicaid	712 (8.1)
Private	5189 (59.0)
Self-pay	273 (3.1)
Other	2221 (25.3)
Geographic region, * *n* (%)	
Northeast	783 (8.9)
Midwest	4824 (54.9)
South	2410 (27.4)
West	701 (8.0)
Puerto Rico	2 (0)
Missing	73 (0.8)
Cohort enrollment time post-index, months ^†^	
Mean ± SD	41 ± 28
Median ± SD	38 (44)
Patients with enrollment time ≥12 months, *n* (%)	7169 (81.5)
Patients with enrollment time ≥24 months, *n* (%)	5864 (66.7)

* Total is greater than 100% because patients could be in multiple categories. Other ethnicity includes records of “Missing” (*n* = 272, 3.1%), “Other ethnicity” (*n* = 258, 2.9%), “Unknown” (*n* = 151, 1.7%), and “Declined” (*n* = 41, 0.5%). Other race includes records of “Other” (*n* = 269, 3.1%), “Missing” (*n* = 240, 2.7%), “Multi-racial” (*n* = 133, 1.5%), “Unknown” (*n* = 61, 0.7%), and “Refused to classify” (*n* = 31, 0.4%). Other insurance type includes records of “Missing” (*n* = 1060, 12.1%), “Other” (*n* = 516, 5.9%), “Unknown” (*n* = 438, 5.0%), and “Other public” (*n* = 207, 2.4%). ^†^ Time from index date to date of disenrollment or end of study period. IQR: interquartile range; oHCM: obstructive hypertrophic cardiomyopathy; and SD: standard deviation.

**Table 2:** Clinical characteristics and treatment management over the study period *.

Patients with oHCM (*n* = 8791)	12-Month Follow-Up, *n* (%)	24-Month Follow-Up, *n* (%)	End of Study Period, *n* (%)
Diagnostic procedures			
Coronary angiography	812 (9.2)	995 (11.3)	1236 (14.1)
Myocardial imaging	6440 (73.3)	7203 (81.9)	7682 (87.4)
Exercise stress testing	1726 (19.6)	2196 (25.0)	2816 (32.0)
Electrocardiography	5041 (57.3)	5887 (67.0)	6456 (73.4)
Inpatient hospitalization	2182 (24.8)	2493 (28.4)	2914 (33.1)
oHCM comorbidities			
Coronary artery disease	3123 (35.5)	3458 (39.3)	3911 (44.5)
Pulmonary hypertension	509 (5.8)	651 (7.4)	1018 (11.6)
Obstructive sleep apnea	1381 (15.7)	1629 (18.5)	1979 (22.5)
Hypertension	6457 (73.5)	6715 (76.4)	7017 (79.8)
Type 2 diabetes	1852 (21.1)	2039 (23.2)	2288 (26.0)
Obesity/overweight	1764 (20.1)	2092 (23.8)	2591 (29.5)
Conduction disorders	1456 (16.6)	1854 (21.1)	2663 (30.3)
Prescription medication			
Beta-blockers	6054 (68.9)	6651 (75.7)	7078 (80.5)
CCBs	2923 (33.2)	3406 (38.7)	4052 (46.1)
ACEIs	1692 (19.2)	2021 (23.0)	2431 (27.7)
ARBs	1063 (12.1)	1289 (14.7)	1657 (18.8)
Anticoagulation/antiplatelet therapy/thrombolytics	4884 (55.6)	5590 (63.6)	6355 (72.3)
Antiarrhythmic therapy			
Disopyramide	151 (1.7)	174 (2.0)	212 (2.4)
Amiodarone	781 (8.9)	907 (10.3)	1141 (13.0)
Surgical procedures			
Septal myectomy	1690 (19.2)	1800 (20.5)	1937 (22.0)
Alcohol septal ablation	34 (0.4)	40 (0.5)	48 (0.6)
Radiofrequency ablation	100 (1.1)	158 (1.8)	282 (3.2)
Pulmonary vein ablation	79 (0.9)	112 (1.3)	196 (2.2)
Other ablation	1363 (15.5)	1492 (17.0)	1718 (19.5)
Coronary revascularization	335 (3.8)	395 (4.5)	506 (5.8)
Valve surgery	746 (8.5)	826 (9.4)	937 (10.7)
Pacemaker	429 (4.9)	532 (6.1)	750 (8.5)
Implantable cardioverter defibrillator	567 (6.4)	708 (8.1)	984 (11.2)
Heart transplantation	14 (0.2)	18 (0.2)	27 (0.3)

ACEI: angiotensin-converting enzyme inhibitor; ARB: angiotensin receptor blocker; CCB: calcium channel blocker; and oHCM: obstructive hypertrophic cardiomyopathy. * Assessed up to the earliest of index date +1 year (for 12-month follow-up), index date +2 years (for 24-month follow-up), and index date to date of disenrollment or 2 July 2019 (end of study period).

**Figure A1:**
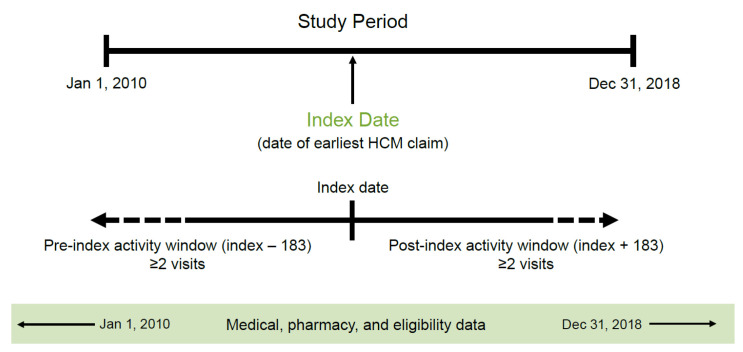
Study time frame. HCM: hypertrophic cardiomyopathy.

## Data Availability

The research data used to support the findings of this study have not been made available because they are proprietary.

## Appendix B

**Table A1:** Demographics and clinical characteristics assessment.

Characteristic	Categories
Age categories, years	18–39, 40–54, 55–64, 65–74, 75+
Ethnicity	Hispanic, non-Hispanic, other ethnicity, declined, unknown, and missing
Race	African American, Asian, White, Hispanic Latino, multi-racial, other, unknown, refused to classify, and missing
Insurance type	Medicare, Medicaid, private, self-pay, other public, other, unknown, and missing
Geographic region	Northeast, Midwest, South, West, Puerto Rico, and missing
Comorbidities	Coronary artery disease, pulmonary hypertension, obstructive sleep apnea, hypertension, type 2 diabetes, obesity/overweight, and conduction disorders
HCM-related outcomes	Atrial fibrillation, congestive heart failure, ventricular and supraventricular arrhythmias, and cardiac arrest
Diagnostic procedures	Coronary angiography, myocardial imaging, exercise/pharmacologic stress testing, electrocardiography, and genetic testing for HCM
Prescription medications	Beta-blockers, CCBs, ACEIs, ARBs, anticoagulation/antiplatelet therapy/thrombolytics, and antiarrhythmic therapy (class I, class III, and other)
Surgical procedures	Septal myectomy, alcohol septal ablation, radiofrequency ablation, pulmonary vein ablation, other ablation, coronary revascularization, valve surgery, pacemaker, implantable cardioverter defibrillator, and heart transplantation
Reintervention	Reintervention is defined as requiring a septal myectomy, alcohol septal ablation, pacemaker, or implantable cardioverter defibrillator after initial septal myectomy or alcohol septal ablation

ACEI: angiotensin-converting enzyme inhibitor; ARB: angiotensin receptor blocker; CCB: calcium channel blocker; and HCM: hypertrophic cardiomyopathy.
